# Evaluation of Accuracy of Preoperative Planning of the Femurofibular Angle in Open-Wedge High Tibial Osteotomy for Mild Medial Knee Osteoarthritis

**DOI:** 10.1155/2021/8813300

**Published:** 2021-02-18

**Authors:** Peizhao Wang, Xiao Wang, Xiaotao Shi, Honglue Tan

**Affiliations:** Department of Knee Surgery, Luoyang Orthopedic-Traumatological Hospital (Henan Orthopedic Hospital), Qiming Southern Road, Luoyang, Henan 471002, China

## Abstract

**Objective:**

The purpose of this study was to evaluate the usefulness of preoperative planning of the femurofibular angle (FFA) in medial open-wedge high tibial osteotomy (OWHTO) for mild medial knee osteoarthritis.

**Methods:**

Thirty-two patients (32 knees) with mild medial knee OA were retrospectively reviewed. The patients underwent preoperative planning of the FFA for OWHTO. For preoperative planning, a full-length weight-bearing X-ray photograph of the lower limb was opened within Adobe Photoshop Software, and a targeted corrective mechanical axis line of the lower limb and its intersecting point at the lateral tibial plateau surface was drawn using rectangle selection and filling tools. A frame, which encircled the tibia and fibula, was created around the predicted osteotomy plane and then rotated until the ankle center was on the targeted mechanical axis line. Subsequently, a distal femoral condyle line and a proximal fibula axis line were drawn, and the angle between the two lines was measured and defined as the femurofibular angle (FFA). During biplane OWHTO, the preoperatively determined FFA was used to complete the correction of the mechanical axis. During follow-up, the postoperative mechanical weight-bearing line (WBL) of the lower limb, the mechanical femorotibial angle (mFTA), and the FFA were measured and compared with the preoperatively determined values.

**Results:**

The mechanical WBL shifted from a preoperative value of 25.36 ± 5.02% to a postoperative value of 56.19 ± 0.10% from the medial border along the mediolateral width of the tibial plateau, and it was 56.57 ± 0.08% at the final follow-up (*P* < 0.01). The preoperatively determined value was 56.25%, and no significant difference was found compared with postoperative week-one and final follow-up values (*P* > 0.05). The mFTA was corrected from a preoperative varus of 4.02 ± 0.63° to a postoperative week-one valgus of 2.37 ± 0.28°, and it had a valgus of 2.48 ± 0.39° at the final follow-up (*P* < 0.01). No significant difference in the valgus was found compared with the postoperative week-one, final follow-up and preoperatively determined valgus of 2.34 ± 0.26° (*P* > 0.05). The postoperative week-one and final follow-up FFAs were 90.34 ± 1.53° and 90.33 ± 1.52°, respectively, and no significant difference was found compared with the preoperatively determined value of 90.12 ± 1.72° and the intraoperative setting value of 90.25 ± 1.67° (*P* > 0.05). All corrected values were within the acceptable range of preoperative planning.

**Conclusion:**

Preoperative planning of the FFA may be useful in OWHTO for patients with mild medial knee OA. Satisfactory correction of the postoperative targeted mechanical axis line of the lower limb can be obtained.

## 1. Introduction

Medial open-wedge high tibial osteotomy (OWHTO) is usually used for the treatment of medial compartment osteoarthritis of the varus knee, and satisfactory long-term clinical outcomes have been reported [1–3]. One of the most important factors for successful OWHTO is that the intraoperative accuracy of the targeted corrective mechanical axis line be consistent with that of preoperative planning, as any small deviation may lead to changes in load distribution over the knee joint, resulting in poor outcomes [3]. Coronal alignment correction using conventional high tibial osteotomy (HTO) approaches, such as the preoperative determination of the required distance of the medial distracted gap and the angle of the wedge bone opening, is not always accurate during OWHTO. Furthermore, other approaches, such as the conventional intraoperative cable method, also show unsatisfactory accuracy, resulting in a mechanical axis line of the lower limb that is outside of the acceptable range [4]. Therefore, it is necessary to identify other approaches to improve the intraoperative correction of the targeted mechanical axis line in modern OWHTO.

Adobe Photoshop Software is an image-processing tool that can modify individual components of an image according to the actual needs of the user [5]. In this study, we used this software for the preoperative simulation and planning of OWHTO for mild medial knee osteoarthritis with poor conservative treatment. After a full-length anteroposterior weight-bearing X-ray photograph of the lower limb was opened within Adobe Photoshop Software, simulated OWHTO was performed based on the targeted mechanical axis line, which was planned preoperatively, and then, the angle between the distal femoral condyle line and the proximal fibula axis line was measured and defined as the femurofibular angle (FFA). During OWHTO, we used the FFA to indirectly monitor the medial gap distraction in real time and ultimately completed the correction of the mechanical axis of the lower limb.

In this study, we used Adobe Photoshop Software (v13.0.1; Adobe Systems Inc., San Jose, CA, USA) for the preoperative planning of FFA for OWHTO in patients with mild medial osteoarthritis of the knee and used the FFA in guiding HTO. The accuracy of the FFA for preoperative planning was evaluated by comparing the preoperatively determined values of the mechanical weight-bearing line (WBL), the mechanical femorotibial angle (mFTA), and the FFA on the long weight-bearing X-ray of the lower limb with those obtained postoperatively.

## 2. Patients and Methods

### 2.1. Inclusion and Exclusion Criteria

This study retrospectively evaluated 36 patients with mild medial knee OA who underwent medial OWHTO from March 2016 to October 2019. The patients agreed to preoperative HTO simulation and measurement of the FFA using Adobe Photoshop Software. The inclusion criteria were as follows: (1) age < 60 years, (2) body mass index (BMI) < 30 kg/m^2^, (3) pain in the medial side only, (4) high level of activity, (5) medial knee osteoarthritis ≤ grade II according to the Kellgren–Lawrence classification, (6) the medial joint space reduced by one-third of the lateral joint space and the joint line convergence angle (JLCA) ≤ 2°, and (7) knee extension loss < 10° and flexion angle > 90°. The exclusion criteria were as follows: (1) HTO in both knees, (2) symptomatic primary patellofemoral OA, (3) history of a knee operation, (4) postoperative follow-up time ≤ 12 months, and (5) incomplete follow-up data.

Based on the aforementioned criteria, two cases had incomplete follow-up data, and two cases had follow-up times < 12 months. Thus, 32 patients were included in this study. There were 14 males and 18 females with a mean age of 53.37 ± 3.82 years (range, 45–59 years). The disease duration was 3.68 ± 1.40 years (range, 1–7 years). The mean BMI was 25.66 ± 2.42 kg/m^2^ (range, 21.09–30.10 kg/m^2^). According to the Kellgren–Lawrence classification, four cases were grade I, and 28 cases were grade II. Patient demographics, as well as preoperative and postoperative limb alignment data, are shown in the Supplemental Files (Tables 1 and 2).

This study was approved by the Ethics Review Committee of Luoyang Orthopedic-Traumatological Hospital (Henan Orthopedic Hospital). Informed consent was obtained from all included subjects. Informed consent was also obtained from a study participant to publish the clinical images in an online publication.

### 2.2. Preoperative FFA Determination

**Figure 1:**
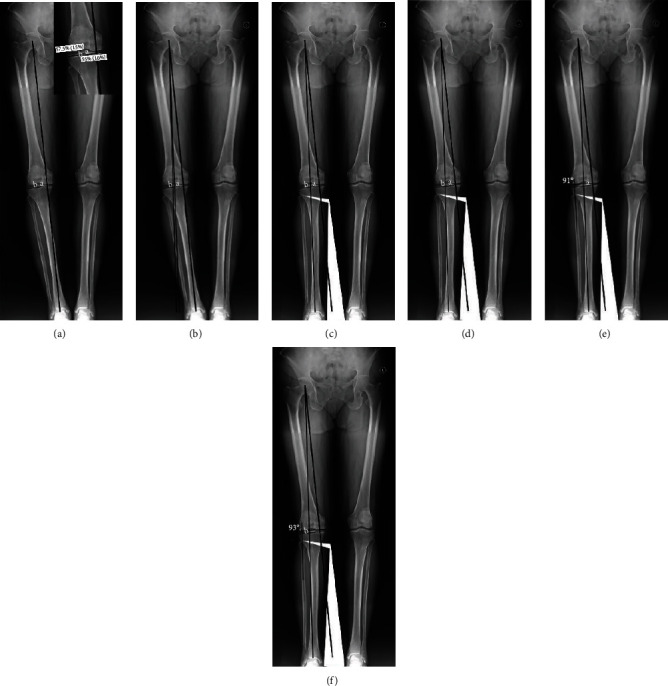
Preoperative planning of FFA. (a) A primary mechanical axis line was drawn from the femoral head center to the ankle joint center on a full-length anteroposterior radiograph of the lower limb. Intersecting point *a* (55%) and intersecting point *b* (57.5%) of the targeted mechanical axis from the medial border of the tibial plateau surface were marked. (b) A corrective mechanical axis line connecting the femoral head center to intersecting point *a* and intersecting point *b* was drawn and extended to the level of the ankle joint (defined as mechanical axis *a* and axis *b*). (c) A frame was created to encircle the predicted osteotomy plan, enclosing the tibia and fibula. The frame was then rotated and moved until the ankle joint center was on the targeted mechanical axis *a*. (d) A similar frame was created and rotated until the ankle joint center was on the targeted mechanical axis *b*. (e) A distal femoral condyle line and a proximal fibula axis line were drawn, and the angle between these two lines was measured and designated as the FFA. The FFA was 91° based on the targeted mechanical axis *a*. (f) The FFA was 93° based on the targeted mechanical axis *b*.

Preoperative FFA determination was performed on a full-length anteroposterior weight-bearing leg X-ray of the lower limb. Firstly, an X-ray image was opened within Adobe Photoshop Software in JPEG format, and a primary mechanical axis was drawn from the center of the femoral head to the center of the ankle joint using rectangle selection and filling tools (Figure 1(a)). Secondly, the intersecting points *a* (55%) and *b* (57.5%) of the targeted mechanical axis line at the lateral tibial plateau surface from the medial border of the tibial plateau surface were calculated and marked according to Jakob principles [6] (Figure 1(a)). Thirdly, two lines were drawn as the targeted mechanical axis *a* and mechanical axis *b*, which, respectively, connected the center of the femoral head with intersecting points *a* and *b* and then extended to the level of the ankle joint using rectangle selection and filling tools (Figure 1(b)). Thereafter, a frame was drawn to encircle the predicted osteotomy plane from the proximal edge of the tibiofibular joint to the predicted medial osteotomy site, which enclosed the tibia and fibula. The frame was selected, rotated, and moved until the center point of the ankle joint was on the targeted mechanical axis *a* and mechanical axis *b*, and the lateral tibial osteotomy site was overlapped point to point (Figures 1(c) and 1(d)). Finally, a distal femoral condyle line and a proximal fibula axis line were drawn using the aforementioned method, and the angle between these two lines was measured and defined as the FFA (Figures 1(e) and 1(f)). Given that the corrected position of the targeted mechanical axis line on the surface of the lateral tibial plateau was expressed as a range, not as a fixed point, the FFA was also expressed as a range (91–93° in this case). This range was used in guiding HTO.

### 2.3. WBL and mFTA Determination

The weight-bearing line (WBL) was defined as a line drawn from the center of the femoral head to the center of the superior articular surface of the talus on long-standing radiographs. The WBL ratio was calculated as the percentage of the distance between the medial edge of the proximal tibia and the point where the WBL intersects the proximal tibia to the width of the tibial plateau (the medial plateau edge was considered 0% and the lateral edge was considered 100%) [7]. The mFTA was defined as the acute angle between the femoral mechanical axis drawn from the center of the femoral head to the center of the knee joint and the tibial mechanical axis drawn from the midpoint of the knee joint to the center of the superior articular surface of the talus [7].

### 2.4. Surgical Technique

**Figure 2:**
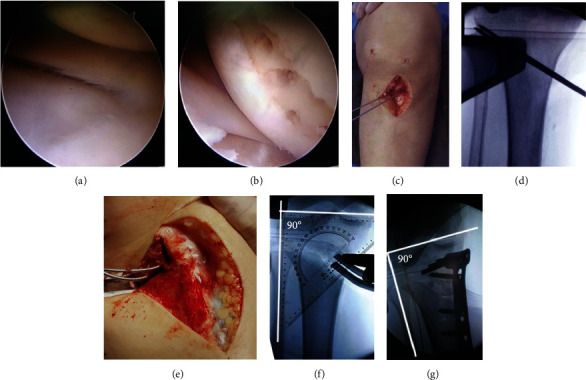
The surgical procedure. (a) Normal cartilage and meniscus of the lateral compartment. (b) Degenerative injury of cartilage and meniscus of the medial compartment. (c) A longitudinal skin incision was made, and the superficial medial collateral ligament, pes anserinus, and patellar ligament were exposed. (d) Two Kirschner wires were drilled parallel into the upper third of the proximal tibiofibular joint. (e) Horizontal osteotomy was performed beneath the two wires, with an angle of 110° to the anterior ascending osteotomy behind the patellar tendon. (f) The osteotomy gap was widened until the intraoperative FFA matched the preoperatively determined FFA. (g) After plate fixation, the FFA was measured again, and the osteotomy gap was filled with bone graft.

The patient was placed in the supine position, and diagnostic arthroscopy was performed to evaluate the joint cartilage and meniscus (Figure 2(a)). Any injuries were treated accordingly (Figure 2(b)). Thereafter, biplanar HTO was performed. Firstly, a longitudinal skin incision ascending approximately 6 cm from the pes anserinus was made, and the superficial medial collateral ligament, pes anserinus, and medial portion of the patellar ligament were exposed. The horizontal osteotomy plane at the level of the pes tendon and the ascending osteotomy plane behind the patellar tendon at an angle of 110° to the horizontal osteotomy plane were marked (Figure 2(c)). Secondly, two 1.5 mm thick Kirschner wires were drilled parallel into the upper third of the proximal tibiofibular joint with ends reaching the lateral tibial cortex (Figure 2(d)). Thirdly, the knee was flexed at an angle of 90°, and horizontal osteotomy was performed with the oscillating saw positioned beneath the two guide wires, creating a 10 mm lateral bone bridge that served as a hinge, and anterior ascending osteotomy was performed with a Kirschner wire drill and narrow bone chisel. With the knee in extension, the osteotomy site was opened with broad flat chisels, and the gap was widened with a bone spreader at the posteromedial corner of the osteotomy (Figure 2(e)). Finally, under radiological observation, the osteotomy gap was stretched until the intraoperative FFA matched the preoperative planning range. To maintain the original posterior tibial slope, the gap in the middle of the osteotomy was two-thirds that of the posterior osteotomy (Figure 2(f)). A TomoFix plate (Synthes GmbH, Solothurn, Switzerland) or another locking plate was used to fix the osteotomy while maintaining the FFA, and the gap was filled with allogenous or autologous bone graft (Figure 2(g)). All surgeries in this study were performed by the same orthopedic team with one senior orthopedic doctor (HLT).

### 2.5. Measurement and Evaluation

The radiological parameters were measured on anteroposterior full-length weight-bearing X-ray radiographs of the lower limb with the patella facing forward using the Picture Archiving Communication System (PACS, PI View STAR, version 5025, INFINITT Healthcare, Seoul, Korea). For comparative evaluation, the WBL ratio and mFTAs were obtained preoperatively and postoperatively. For mFTA, the varus angle was recorded as positive (+), and the valgus angle was recorded as negative (−). The preoperatively determined FFA and intraoperatively and postoperatively measured FFA were compared to evaluate its effectiveness in guiding OWHTO for mild medial knee OA. All radiographic measurements were performed independently by two orthopedic surgeons (XTS and HLT) and taken twice after an interval of 2 weeks.

### 2.6. Statistical Analysis

All radiological measurements are presented as the mean ± standard deviation (SD). The Kolmogorov–Smirnov test was performed to determine whether the data conformed to the normal distribution. The paired *t*-test was used to compare preoperative and postoperative variables with a normal distribution. The Wilcoxon test was used for continuous variables without a normal distribution. All statistical analyses were performed using SPSS 19.0 Software (IBM, Armonk, NY, USA), and a *P* value < 0.05 was considered statistically significant. The intraobserver and interobserver reliabilities of the radiological measurements were assessed by determining the intraclass correlation coefficient (ICC), which ranged from 0 to 1, with 1 indicating perfect reliability and 0 indicating unreliability [7]. In this study, the intraobserver agreements ranged from 0.90 to 0.95, and the interobserver agreements ranged from 0.88 to 0.93, indicating high reliability and excellent measurement reproducibility. The ICC results are shown in the Supplemental Files (Table 2).

## 3. Results

**Figure 3:**
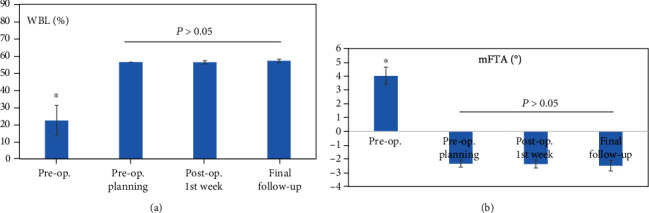
The mechanical WBL and mFTA before and after surgery. ^∗^*P* < 0.01 compared with pre-op. planning, post-op. 1^st^ week, and final follow-up (Wilcoxon test).

**Figure 4:**
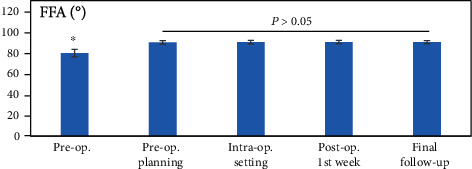
The FFA before and after surgery. ^∗^*P* < 0.01 compared with pre-op. planning, intraoperative, post-op.1^st^ week, and final follow-up (Wilcoxon test).

**Figure 5:**
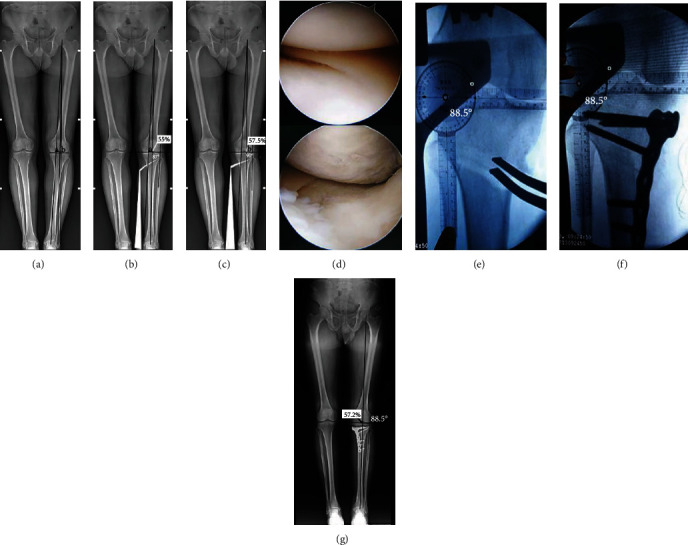
A 56-year-old female patient with medial compartment osteoarthritis of the left knee underwent OWHTO. (a) The primary mechanical axis and targeted corrective mechanical axis *a* (55%) and *b* (57.5%) from the medial border of the tibial plateau surface on the full-length anteroposterior radiograph of the lower limb were marked. (b) The preoperative simulation of OWHTO according to the mechanical axis *a* and the FFA was 87°. (c) The simulation of OWHTO according to the mechanical axis *b* and the FFA was 90°. (d) Arthroscopy showed an intact meniscus and cartilage in the lateral compartment and degeneration in the medial compartment. (e) Intraoperative osteotomy was carried out until the FFA was in the range of preoperative planning of 87–90° (88.5° in this case). (f) After fixing the plate in position, the FFA was measured again, and it was 88.5°. (g) The FFA was 88.5° and the mechanical WBL was 57.2% at 18-month follow-up, which is consistent with the values of preoperative planning and intraoperative setting.

The mean follow-up time was 37.63 ± 11.30 months (range, 18–58 months). The mechanical WBL shifted from a preoperative value of 25.36 ± 5.02% (range, 18–35%) to a postoperative value of 56.19 ± 0.10% (range, 55–58%) from the medial border along the mediolateral width of the tibial plateau, and it was 56.57 ± 0.08% (range, 56–58%) at the final follow-up (*P* < 0.01). The preoperatively planned value was 56.25% (acceptable range, 55–57.5%), and no significant difference was found compared with postoperative week-one and final follow-up values (*P* > 0.05) (Figure 3(a)). The mFTA was corrected from a preoperative varus of 4.02 ± 0.63° (range, 2.5–5°) to a postoperative week-one valgus of 2.37 ± 0.28° (range, 2–3°), and it had a valgus of 2.48 ± 0.39° (range, 2–3.5°) at the final follow-up (*P* < 0.01). No significant difference in the valgus was found compared with the postoperative week-one, final follow-up and preoperatively planned valgus of 2.34 ± 0.26° (acceptable range, 1.9–2.8°) (*P* > 0.05) (Figure 3(b)). The postoperative week-one and final follow-up FFAs were 90.34 ± 1.53° (range, 87–93°) and 90.33 ± 1.52° (range, 86–92°), respectively, and no significant differences were found compared with the preoperatively planned value of 90.12 ± 1.72° (acceptable range, 85.75–92.25°) and the intraoperative setting value of 90.25 ± 1.67° (range, 85–92°) (*P* > 0.05) (Figure 4). The deviation of WBL, mFTA, and FFA between the preoperatively planned value and the final follow-up value was 0.004 ± 0.007%, 0.148 ± 0.107°, and 0.211 ± 0.036°, respectively. All corrected values were within the acceptable range of preoperative planning. A typical case is seen in (Figure 5).

## 4. Discussion

Accurate preoperative planning and intraoperative correction of varus deformities are the most important criteria for successful OWHTO [3, 8]. A full-length weight-bearing X-ray of the lower limb is usually obtained for the preoperative planning of the targeted corrective mechanical axis line, the required distance of the medial distracted gap, and the angle of the wedge bone opening. Successful intraoperative osteotomy requires the corrective mechanical axis line, gap distance, and angle degree to be consistent with the preoperatively determined values. As for the significance of these three indexes in the accurate guidance of OWHTO, inaccurate measurements are commonly encountered before and during surgery. For example, an inaccuracy in the distance of the preoperative X-ray projection may lead to an incorrect measurement of the preoperatively determined distraction gap. Although the medial distracted gap distance and the angle degree of the osteotomy could be calculated precisely, bone loss due to the repeated movement of the saw blade and the compression of the posteromedial bone at the osteotomy site caused by osteoporosis and the bone spreader may increase the gap distance and angle degree, thereby causing undercorrection or overcorrection during surgery [9]. The targeted mechanical axis line can be intraoperatively monitored in real time with a radiopaque rod or an electrocautery cord; however, this can lead to unsatisfactory outcomes and expose the surgeon and patient to X-ray radiation due to the increased time required to identify the hip and ankle joint centers and to verify the degree of correction [10, 11]. A computer navigation-based technique for HTO has recently been reported, and its accuracy and superiority have been confirmed [12]. However, digital registration of lower limbs greatly prolongs the operation time, and its high cost prohibits its routine use by most hospitals [13].

Adobe Photoshop Software is well known for its extensive drawing and editing functions, and it can effectively edit an image according to the needs of the user [5]. In this study, we used it for the preoperative simulation of medial OWHTO for mild medial knee OA, which accurately displayed the coronal appearance of the entire corrected lower limb and the tilt degree of the joint line after OWHTO. After using this software for the preoperative planning of OWHTO, the FFA was measured and used to indirectly monitor the correction of the knee deformity and the targeted mechanical axis transfer in real time during surgery. As demonstrated by the radiological results, the postoperative values of WBL, mFTA, and FFA were within the acceptable range of preoperative planning, and no significant differences in the values were found before and after surgery.

Postoperative limb alignment is an important factor for successful OWHTO. However, there is no consensus on the crossing point of the targeted corrective mechanical axis line on the tibial plateau surface. A recent study reported that the targeted WBL should take into consideration the degree of osteoarthritis of the knee; it showed that one uniform alignment does not exist, and postoperative alignment should be tailored to the patient's needs [14]. In our clinical practice, we obtain the crossing point of the WBL at the lateral tibial plateau surface according to the degree of medial compartment osteoarthritis, that is, if 0% is taken as the center of the knee joint and 100% is taken as the lateral edge of the plateau, the targeted WBL will run through the 10–15% position for one-third loss of the medial cartilage thickness, the 20–25% position for two-thirds loss, and the 30–35% position for total loss of the medial cartilage thickness [6]. In this study, patients with mild medial knee OA were selected, and the loss of the medial cartilage thickness was one-third of the lateral compartment; thus, the mechanical axis line of the lower limb was corrected to the 10–15% position from the center of the knee joint (55–57.5% position from the medial border of the tibial plateau surface). Based on this principle, the preoperative planning FFA was expressed as a range; and using the FFA for OWHTO, it was acceptable for the postoperative targeted mechanical axis line being within the range of preoperative planning.

For degenerated medial knee OA with the loss of joint space, despite accurate preoperative planning and careful surgical techniques during OWHTO, not all patients can receive the preoperatively planned alignment [15]. One of the main reasons is the postoperative overcorrection of the deformity caused by preoperative medial soft tissue laxity, which is often represented by the JLCA formed by two articular tangential lines of the distal femur and the proximal tibia [7, 16]. Therefore, preoperative correction planning should consider the JLCA. For medial OWHTO, target point shifting within the coronal hypomochlion point (weight-bearing line passing through 57.5% of the total width of the proximal tibia; corresponding mechanical tibiofemoral angle (mTFA), 2°) could be considered to prevent overcorrection [14]. A biomechanical study has reported that overcorrection beyond 3° of the mechanical valgus has no advantage for unloading the medial peak pressure [17]. Other studies also showed that even a neutral mechanical alignment (mTFA = 0°) can decrease the mean contact pressure of the medial compartment [18]. According to these results, satisfactory decompression of the medial knee compartment can be obtained when the mechanical lower limb alignment is corrected to neutral and 3° of the valgus, and excessive valgus correction can be avoided. To exclude the obvious influence of latent medial soft tissue laxity on the postoperative mechanical alignment correction, patients with medial JLCA ≤ 2° were included in this study. For these patients, the loss of the medial cartilage thickness was one-third of the lateral compartment, and the mechanical axis line of the lower limb was corrected to 55–57.5% from the medial border of the tibial plateau surface [6]. At this time, the mFTA was maintained at an averaged 2.06 ± 0.37° of the valgus in our study, which means that medial compartment decompression was achieved. One study showed a linear change of JLCA in a range of 0° to 5° of the valgus, which started when the postoperative long-leg axis was corrected beyond 2° of the mFTA, and the authors recommended to plan realignment for medial OWHTO at a maximum of 2° of the valgus [19]. Thus, JLCA will not cause obvious overcorrection of the lower limb alignment owing to 2.06 ± 0.37° of the valgus of mFTA in our study. Accordingly, using the preoperatively planned FFA to guide the accurate correction of the mechanical axis during medial OWHTO, the postoperative FFA did not change, that is to say that the preoperatively planned FFA can be used to guide OWHTO. The consistency of the measured values of the relevant quantitative index of the mechanical axis correction before and after surgery also confirmed the validity of the preoperatively planned FFA as guidance for OWHTO in patients with mild medial knee OA.

Using preoperative FFA for successful medial OWHTO requires careful consideration of several criteria. Firstly, surgical indications should be strictly selected for medial knee OA, as the medial joint space is reduced by one-third of the lateral joint space and JLCA ≤ 2°. Secondly, preoperative long-standing anteroposterior radiographs of the lower limbs with the patellae facing forward should be obtained. Thirdly, when using Adobe Photoshop Software for OWHTO simulation, the predetermined intersection of the targeted mechanical axis line should be expressed as a range on the lateral tibial plateau surface. Therefore, the measured FFA should also be expressed as a range, and it should be acceptable for the intraoperative FFA within the preoperative values after completion of the osteotomy and gap distraction. Finally, the intraoperative FFA should be consistent with preoperative planning. Therefore, the knee joint should be maintained in a neutral position after osteotomy, whereas the whole knee joint and proximal fibula should be clearly visible by fluoroscopy.

There are several limitations in this study. Firstly, this is a retrospective and nonrandomized-controlled study, indicating that patient selection and result bias may exist. Prospective randomized-controlled studies are needed in which groups are compared by the traditional method such as the intraoperative cable method for mechanical axis correction. Secondly, our population size was small and the follow-up period was short. Therefore, further studies are needed with a greater number of patients and a longer follow-up time. Thirdly, this study did not include clinical evaluation, and further studies will be required to clarify the lower limb realignment with optimal long-term clinical results.

## 5. Conclusions

Based on the full-length weight-bearing X-ray of the lower limb and the targeted corrective mechanical axis line, FFA after OWHTO can be preoperatively determined by Adobe Photoshop Software. According to the planned FFA for guiding OWHTO in mild medial knee OA, the targeted corrective mechanical axis line after surgery can be obtained. The lack of significant differences between the preoperatively determined and postoperatively achieved values supports the preoperative planning of FFA for OWHTO.

## Data Availability

The datasets used and/or analysed during the current study are available from the corresponding author on reasonable request.

## Abbreviations

OA: Osteoarthritis
FFA: Femurofibular angle
OWHTO: Open-wedge high tibial osteotomy
WBL: Weight-bearing line
mFTA: Mechanical femorotibial angle
BMI: Body mass index
SD: Standard deviation
JLCA: Joint line convergence angle.

## References

[1] Duivenvoorden T., van Diggele P., Reijman M. (2017). Adverse events and survival after closing- and opening-wedge high tibial osteotomy: a comparative study of 412 patients. *Knee Surgery Sports Traumatology Arthroscopy*.

[2] Giuseffi S. A., Replogle W. H., Shelton W. R. (2015). Opening-wedge high tibial osteotomy: review of 100 consecutive cases. *Arthroscopy*.

[3] Van den Bempt M., Van Genechten W., Claes T., Claes S. (2016). How accurately does high tibial osteotomy correct the mechanical axis of an arthritic varus knee? A systematic review. *The Knee*.

[4] Hankemeier S., Hufner T., Wang G. (2006). Navigated openwedge high tibial osteotomy: advantages and disadvantages compared to the conventional technique in a cadaver study. *Knee Surgery Sports Traumatology Arthroscopy*.

[5] Kiranantawat K., Nguyen A. H. (2015). Asian rhinoplasty: preoperative simulation and planning using Adobe Photoshop. *Seminars in Plastic Surgery*.

[6] Marti C. B., Gautier E., Wachtl S. W., Jakob R. P. (2004). Accuracy of frontal and sagittal plane correction in open-wedge high tibial osteotomy. *Arthroscopy*.

[7] Takagawa S., Kobayashi N., Yukizawa Y., Oishi T., Tsuji M., Inaba Y. (2020). Preoperative soft tissue laxity around knee was associated with less accurate alignment correction after hybrid closed-wedge high tibial osteotomy. *Knee Surgery Sports Traumatology Arthroscopy*.

[8] Kim H.-J., Lee H.-J., Shin J.-Y., Park K.-H., Min S.-G., Kyung H.-S. (2017). Preoperative planning using the picture archiving and communication system technique in high tibial osteotomy. *Journal of Orthopaedic Surgery*.

[9] Sabharwal S., Zhao C. (2008). Assessment of lower limb alignment: supine fluoroscopy compared with a standing full-length radiograph. *Journal of Bone and Joint Surgery (American)*.

[10] Schröter S., Ihle C., Mueller J., Lobenhoffer P., Stöckle U., van Heerwaarden R. (2013). Digital planning of high tibial osteotomy. Interrater reliability by using two different software. *Knee Surgery, Sports Traumatology, Arthroscopy*.

[11] Yoon S. D., Zhang G., Kim H. J., Lee B. J., Kyung H. S. (2016). Comparison of cable method and miniaci method using picture archiving and communication system in preoperative planning for open wedge high tibial osteotomy. *Knee Surgery & Related Research*.

[12] Blakeney W. G., Khan R. J. K., Wall S. J. (2011). Computer-assisted techniques versus conventional guides for component alignment in total knee arthroplasty. *Journal of Bone and Joint Surgery*.

[13] Kyung B. S., Kim J. G., Jang K. M. (2013). Are navigation systems accurate enough to predict the correction angle during high tibial osteotomy?. *The American Journal of Sports Medicine*.

[14] Feucht M. J., Minzlaff P., Saier T. (2014). Degree of axis correction in valgus high tibial osteotomy: proposal of an individualised approach. *International Orthopaedics*.

[15] Gaasbeek R. D. A., Nicolaas L., Rijnberg W. J., van Loon C. J. M., van Kampen A. (2010). Correction accuracy and collateral laxity in open versus closed wedge high tibial osteotomy. A one-year randomised controlled study. *International Orthopaedics*.

[16] Ogawa H., Matsumoto K., Ogawa T., Takeuchi K., Akiyama H. (2016). Preoperative varus laxity correlates with overcorrection in medial opening wedge high tibial osteotomy. *Archives of Orthopaedic and Traumatic Surgery*.

[17] Mina C., Garrett W. E., Pietrobon R., Glisson R., Higgins L. (2017). High tibial osteotomy for unloading osteochondral defects in the medial compartment of the knee. *The American Journal of Sports Medicine*.

[18] Riegger-Krugh C., Gerhart T. N., Powers W. R., Hayes W. C. (1998). Tibiofemoral contact pressures in degenerative joint disease. *Clinical Orthopaedics and Related Research*.

[19] Heijens E., Kornherr P., Meister C. (2016). The coronal hypomochlion. *The Bone & Joint Journal*.

