# Supplementary figures and images for: Evaluation of Accuracy of Preoperative Planning of the Femurofibular Angle in Open-Wedge High Tibial Osteotomy for Mild Medial Knee Osteoarthritis
Source: Biomed Res Int. 2021 Feb 18;2021:8813300. doi: 10.1155/2021/8813300 (PMC7985241; doi:10.1155/2021/8813300)

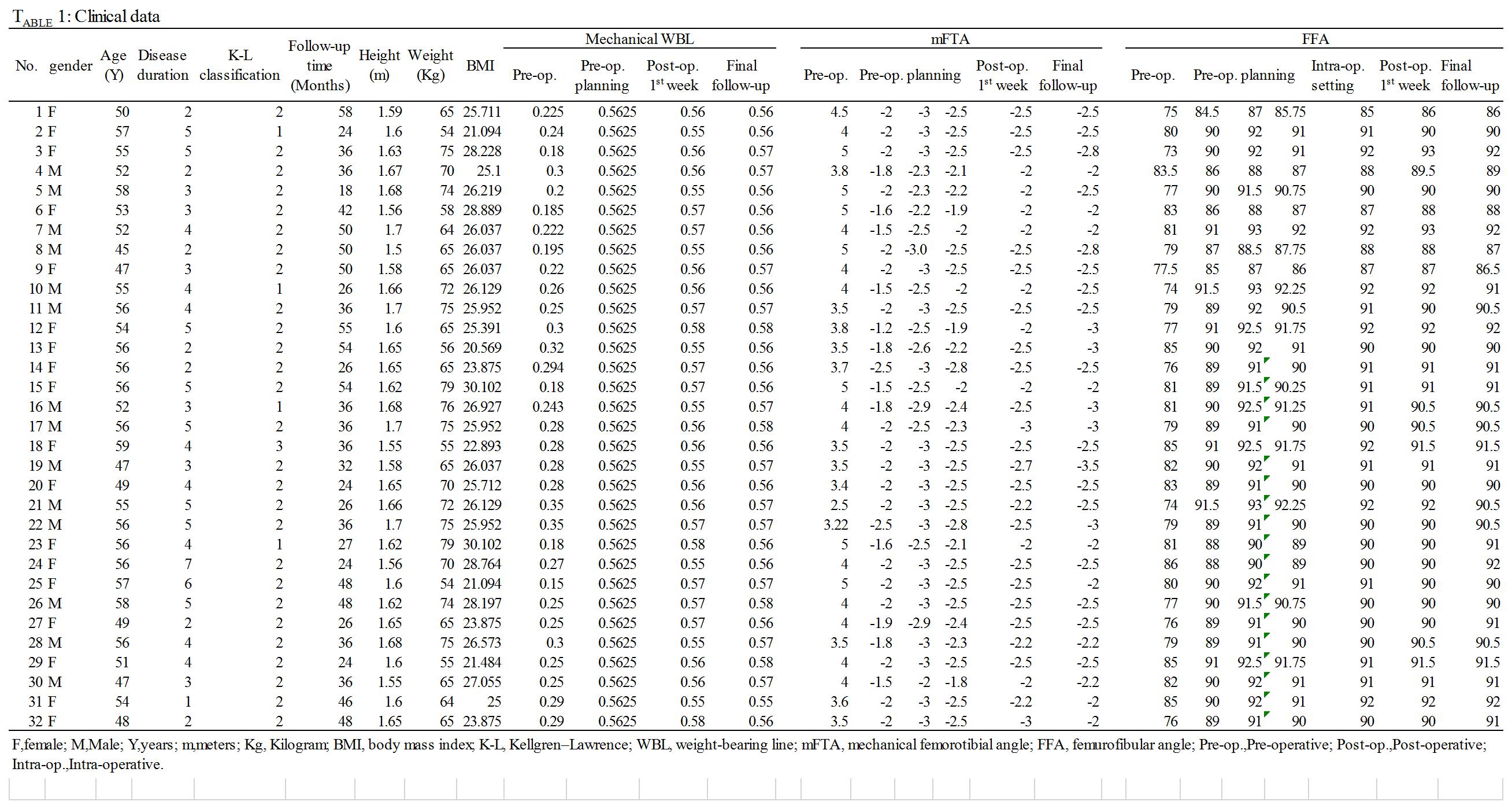

Supplement: Supplementary Materials — Clinical data are provided in the supplementary tables [file 8813300.f1.zip › 8813300.f1.jpg]

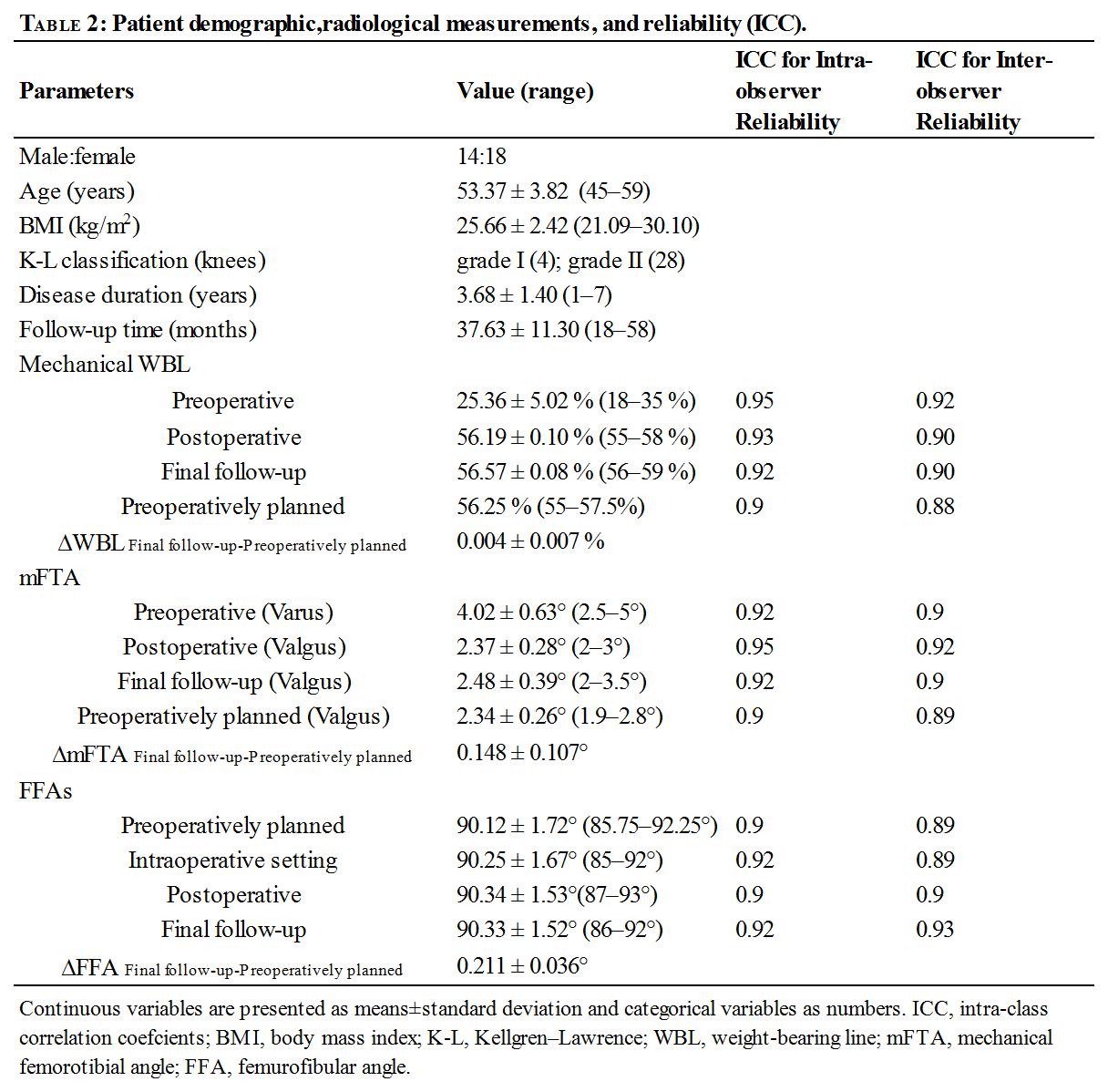

Supplement: Supplementary Materials — Clinical data are provided in the supplementary tables [file 8813300.f1.zip › 8813300.f2.jpg]
